# Nature of Heat and Thermal Energy: From Caloric to Carnot’s Reflections, to Entropy, Exergy, Entransy and Beyond

**DOI:** 10.3390/e20080584

**Published:** 2018-08-07

**Authors:** Milivoje M. Kostic

**Affiliations:** Department of Mechanical Engineering, Northern Illinois University, DeKalb, IL 60115, USA; kostic@niu.edu

**Keywords:** entransy, entropy, exergy, heat, thermal energy, Carnot work potential

## Abstract

The nature of thermal phenomena is still elusive and sometimes misconstrued. Starting from Lavoisier, who presumed that caloric as a weightless substance is conserved, to Sadi Carnot who erroneously assumed that work is extracted while caloric is conserved, to modern day researchers who argue that thermal energy is an indistinguishable part of internal energy, to the generalization of entropy and challengers of the Second Law of thermodynamics, the relevant thermal concepts are critically discussed here. Original reflections about the nature of thermo-mechanical energy transfer, classical and generalized entropy, exergy, and new entransy concept are reasoned and put in historical and contemporary contexts, with the objective of promoting further constructive debates and hopefully resolve some critical issues within the subtle thermal landscape.

## 1. Introduction—From Caloric to Carnot’s Reflections

There is a need to “shed more light onto dissipative heat”. It is the goal of this treatise to contribute to that aspiration. Richard Feynman once stated, “It is important to realize that in physics today, we have no knowledge what energy is” [1]. This statement has a deeper connotation, since we tend to simplify, pre-judge, and proclaim definite meanings of the fundamental concepts, or to discredit new ones. Heat and thermal energy are more subtle and elusive than many other forms of energies. Nature is, and so is heat, what it is, no more and no less. In fact, all other forms of energies are ultimately dissipating in thermal heat, the omnipresent and universal phenomena, quantified with perpetual and irreversible generation of thermal displacement, i.e., entropy. Science and technology have evolved over time on many scales and levels, so that we now have the advantage to look at related historical developments more comprehensively than the pioneers. The fundamental laws of thermodynamics, and especially the issues related to thermal energy and entropy, including the Second law challenges, have been of primary interest and the topic of this author’s past presentations and recent writings. Long-contemplated reflections on some critical issues of thermoscience concepts, from unpublished presentations and selected citations with updates, are presented here to hopefully contribute to further discussions and encourage due debate.

There are many puzzling issues surrounding thermodynamics and the nature of heat, including subtle definitions and ambiguous meanings of very fundamental concepts. In modern times, there is a tendency by some authors to unduly discredit thermal energy as being indistinguishable from other internal energy types. Romer [2] argues that “Heat is not a noun”, and proposes to remove it from the dictionary. Ben-Naim [3] titles his book “A Farewell to Entropy”, while Leff [4] in a series of articles entitled “Removing the Mystery of Entropy and Thermodynamics”, argues surprisingly, that “Entropy can be introduced and understood without ever mentioning heat engines”, and against the “thermal energy” concept in favor of more modern and well-defined “internal energy”.

The nature of heat was intriguing since the introduction of caloric and still is an elusive concept. Lavoisier proposed that “heat is a subtle, weightless substance called caloric”, with the assumption of “conservation of caloric”. The “caloric” was not obviously conserved during dissipative “heat generation” processes, such as drilling, and similarly, so that “caloric theory” has been discredited, regardless of ingenious developments. Caloric was not also conserved in heat engines, as mistakenly assumed by Sadi Carnot [5]. In fact, the caloric theory is invaluable to modern calorimetric property measurements, and it should only be objectively re-evaluated and augmented with contemporary thermal developments.

Sadi Carnot (1824) laid ingenious foundations for the Second Law of thermodynamics and discovery of entropy before the First law of energy conservation was even known (Joule, 1843, and Helmholtz, 1847), and long before thermodynamic concepts were established in the second half of the nineteenth century. In the historical context, it is hard to comprehend now, how Carnot then, at age 28, ingeniously and thoroughly explained the critical concepts of reversible thermo-mechanical processes and the limits of converting heat to work at inception of the heat engines’ era, when the nature of heat was not fully understood. Note that Carnot erroneously assumed that the same caloric (heat) passes through the engine and extracts (produces) work by lowering its temperature, similar to how the same water flow passes through the water-wheel and produces work by lowering its elevation potential. This error (violating energy conservation), considering the knowledge at the time, in no way diminishes Carnot’s ingenious reasoning and conclusions about limiting, reversible processes and its accurate limitations of heat to work conversion [6].

## 2. Nature of Thermo—Mechanical Energy Transfer

**Figure 1:**
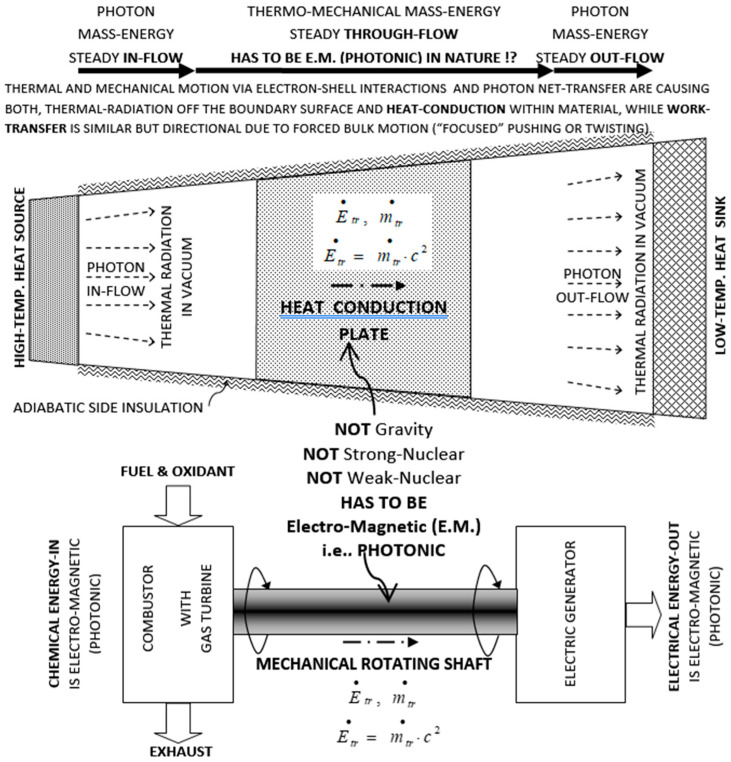
Electromagnetic nature of thermo-mechanical mass-energy transfer due to photon diffusive re-emission and propagation. Based on atomic electron-shell interactions and the Einstein mass-energy equivalence, during “believed-massless” heat conduction or mechanical work transfer there has to be electromagnetic, photonic mass-energy propagation through involved material structures from a mass-energy source to a sink system. Steady-state, mass-energy transfer is depicted through heat conduction plate (at Figure top) and rotating shaft (at Figure bottom). Energy transfer (i.e., Einstein’s mass-energy equivalency transfer, ${\dot{E}}_{tr}={\dot{m}}_{tr}c^{2}$) has to be electromagnetic by photon transfer, either as photon electromagnetic waves on-long range through space/vacuum (${\dot{Q}}_{rad}={\dot{m}}_{rad}c^{2})$, or photon “on-contact” transfer (annihilation/reemission) within material structures, e.g., through heat conduction plate (at Figure top) and turbine shaft work (at Figure bottom), since it is neither gravitational nor nuclear (strong or weak) interaction. Otherwise, Einstein’s mass-energy equivalency and the fundamental force/interactions will be violated. Thermal conduction is due to chaotic thermal electron-shell collisions and may be enhanced by free-electrons or crystal-lattice structure vibration (phonons), both phenomena due to underlining photon propagation (similar to electro-chemical phenomena). The mechanical work transfer is due to electron-shell directional pushing/twisting as the most efficient (“focused”) energy transfer (i.e., mechanical super conductor). If it is fully investigated and understood, it has potential for development of hybrid synthetic-materials with superior thermal conductivity such as diamond and others, for critical and new applications [8].

It is widely believed that thermal heat conduction and mechanical work transfer are “massless” phenomena. However, based on existing observations of atomic electron-shell interactions and well-established phenomena and theories, including Einstein’s mass-energy equivalence [7] and thermal radiation, it is reasoned here that for a conduction heat transfer (e.g., through a wall) or mechanical work transfer (e.g., a rotating shaft), there has to be underlying electromagnetic energy transfer (i.e., via photon “on-contact” diffusive annihilation/reemission and propagation) and commensurate mass-transfer (*m* = *E/c*^2^) through material systems involved, from a mass-energy source to a sink system, as depicted on Figure 1, and detailed in its caption. Ironically, Lavoisier was correct that caloric is a substance, but not weightless. More details were presented [8] and will be published after updates are finalized.

## 3. Nature of Heat and Thermal Energy

**Figure 2:**
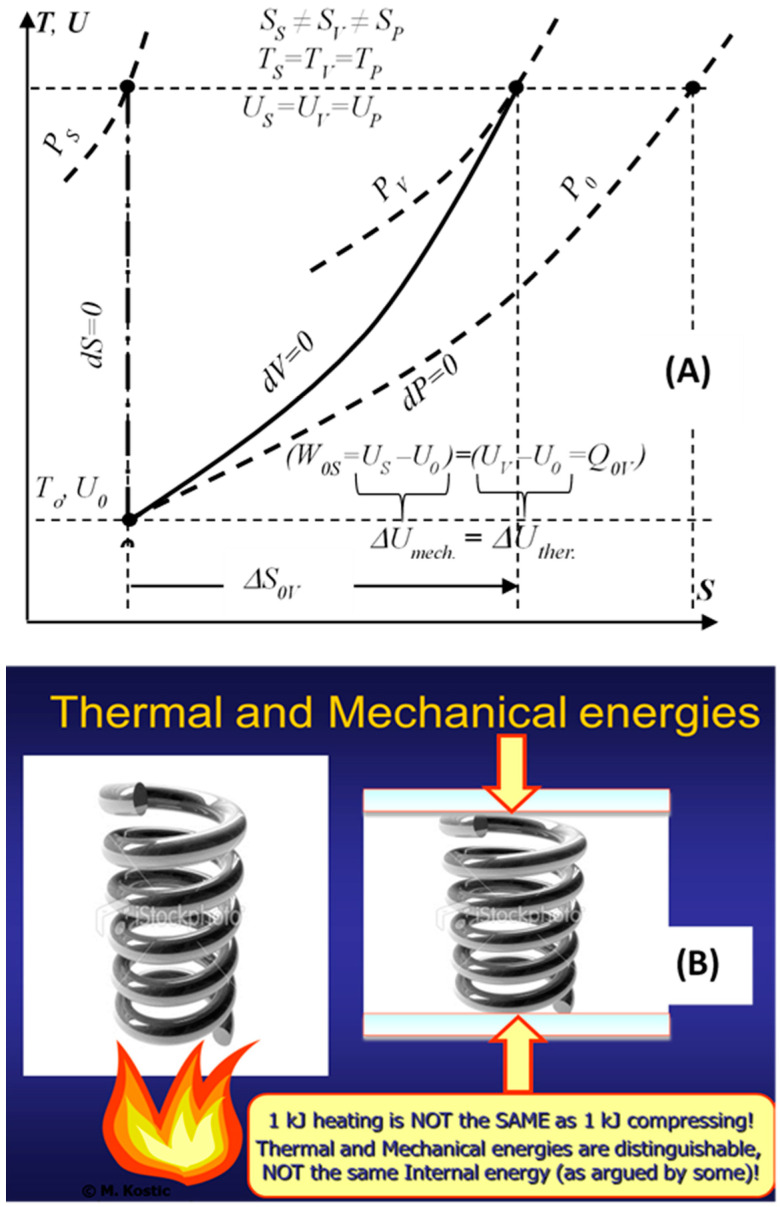
Thermal and mechanical internal energies are distinguishable parts of the thermodynamic internal energy, the former increasing the thermal and the latter increasing the mechanical part of the internal energy, resulting in different states, regardless that the internal energies are the same, as illustrated by 1 kJ heating or 1 kJ compressing of ideal gas (**A**), or a spring (**B**).

Denying the existence of thermal energy is the same as denying the existence of its transfer (heat transfer) [9,10]. Some consider the Thermodynamic internal energy to be the thermal energy, although the former represents all energy types stored as the kinetic and potential energies of the constituent microstructure, namely, the thermal and mechanical-elastic energies in simple compressible substances, in addition to the chemical and nuclear internal energies. In more complex system structure there are more energy types. The stored system heat increases the system thermal energy that is distinguished from the system internal, mechanical (elastic) energy. For example, the heating or compressing an ideal gas with the same amount of energy will result in the same temperatures and internal energies, but different states, with different volumes and entropies, and similar for other material substances, see Figure 2 [9,10]. Reversible heat transfer and caloric heat transfer, without work interactions, are introduced as limiting processes of heat-work interactions. It is reasoned and deduced, that the thermal energy is distinguishable, regardless of its coupling with the other internal energy forms, and thus paving the way to further illuminate other critical concepts, including thermodynamic entropy, entropy-generation, the Second law of energy degradation, and the new entransy concept.

Heat is a unique and universal concept representing energy transfer of thermal random-motion and its interactions, while all other energy transfers are classified as different types of work. The “thermal energy” or “stored heat” represents stored energy of relevant thermal motion and interactions due to thermal heat transfer or heat-generation, i.e., dissipation-conversion of all other energy types to thermal heat. The term “thermal heat” represents here the holistic meaning of both, the heat as transfer of the stored thermal energy and the stored thermal energy itself.

**Figure 3:**
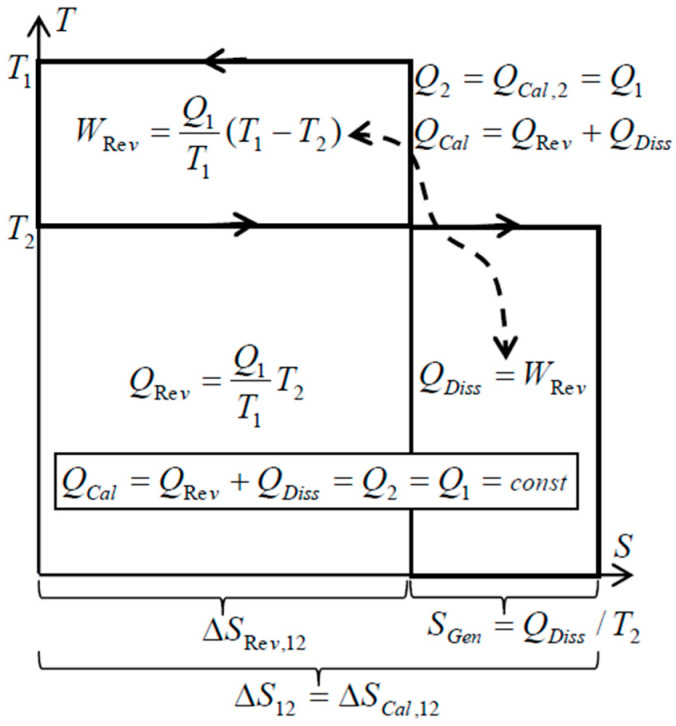
During a spontaneous caloric heat transfer process between two thermal reservoirs, the work potential, *W_Rev_*, is completely dissipated into heat at a lower temperature, *Q_Diss_*, which after being added to the reduced reversible heat at lower temperature, *Q_Rev_*, will result in conserved heat or thermal energy, *Q_Cal_* = *Q_Rev_* + *Q_Diss_* = *constant*, with increased, generated entropy in the amount of dissipated work potential per relevant absolute temperature [9,10].

There is an important peculiarity about spontaneous heat transfer processes such as within simple heat exchangers (without forced flow work): no heat conversion to work like in heat engine, and no other heat generation from work dissipation, but only the Carnot’s “thermal work-potential dissipation” to heat itself at lower temperature—resulting in the conservation of thermal energy, i.e., conservation of thermal heat [9,10,11]. Just like the original caloric—the thermal energy is conserved on its own, but spontaneously degraded to a lower temperature (dissipation of thermal work-potential, and dissipation of entransy), since it cannot be spontaneously reversed back to higher temperatures. Such processes, without work interactions, are called here as “caloric processes” or “caloric heat transfer”. The “reversible heat transfer“, could also be defined as limiting process when the heat source and heat sink are at a finite temperature difference, accomplished by an ideal Carnot cycle so that thermal work-potential is extracted (instead of being dissipated into heat, such as in the above “caloric processes”), while adjusting temperature levels so that “reversible heat transfer” takes place at infinitesimally small temperature difference at each temperature level (dT→0) [9,10,11], see Figure 3. Therefore, the Carnot principle defines both: thermal work-potential and reversible heat transfer.

The stored system heat increases the system’s “thermal energy” and entropy, the former is distinguished from the other internal-energy types (e.g., mechanical elastic energy). A related manuscript is being updated, to quantify the thermal energy within the system internal energy [10].

## 4. Entropy: Thermal (Chaotic-Dynamic) Displacement

What is the underlying nature of “entropy” and why does it always increase? Why is entropy so intriguing and mysterious, unique and universal, as if it is a miraculous property of natural, material systems? How does it encompass and quantify all processes at all natural space and time scales, governed by the Second law of thermodynamics? And many other elusive and debatable issues, as if entropy is among the deepest unresolved mysteries in nature, defying our common sense [11]. Entropy is the most used and often abused concept in science, but also in philosophy and society. Further confusions are produced by some attempts to generalize entropy with similar, but not the same concepts in other disciplines. Von Neumann once remarked that “whoever uses the term ‘entropy’ in a discussion always wins since no one knows what entropy really is, so in a debate one always has the advantage”.

Carnot paved the way for his followers to define and prove that entropy is a state function, a material property conserved in ideal, reversible cycles (Clausius Equality—definition of entropy property), that entropy could not be destroyed since it will imply more efficient than ideal cycles (and ideal processes), but is always generated (locally and globally, thus overall increased) due to dissipation of any and all work potentials to heat, causing generation of entropy in irreversible cycles (Clausius Inequality—definition of entropy generation); thereby, quantifying all reversible and irreversible processes and providing generalization of the Second law of thermodynamics [5,6]. Note that Carnot erroneously assumed that the same caloric (heat) passes through the engine and extracts (produces) work by lowering its temperature, similar to how the same water flow passes through the water-wheel and produces work by lowering its elevation potential. This error, considering the knowledge at the time, in no way diminishes Carnot’s ingenious reasoning and conclusions about limiting, reversible processes and its accurate limitations of heat to work conversion [6].

**Figure 4:**
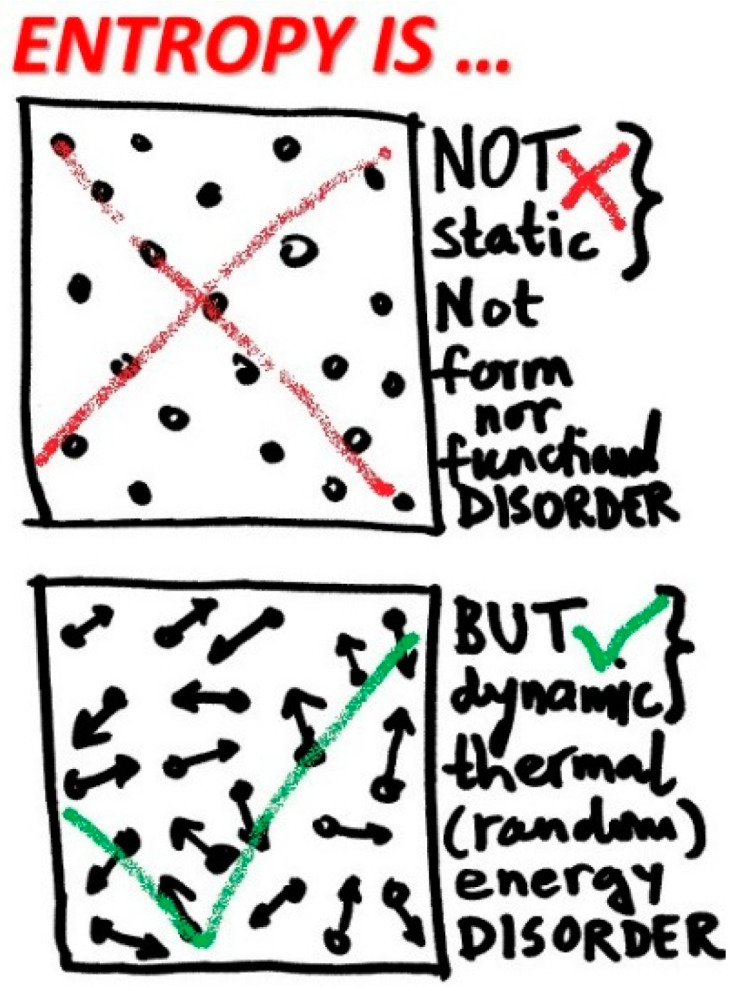
Entropy is not a space disorder, nor form, nor functional disorder. Entropy is a thermal motion disorder. No thermal motion, no entropy! Expanding entropy to any type of disorder or information is a source of many misconceptions.

Entropy is related to thermal motion of a system microstructure, the latter gives rise to all thermal phenomena and related properties, namely, temperature, thermal or heat capacity, thermal energy and entropy, among others, see Figure 4. Due to conversion of thermal energy to other energy forms, such as mechanical work in heat engine, and also spontaneous and unavoidable dissipation of all other energy forms to thermal energy via so called heat generation, additional issues and sometimes confusions arise. However, entropy is a well-defined material system macro-property, precisely measured, and tabulated and/or correlated, for practical use in engineering and science. Entropy should be further reasoned, refined and explained for what it is, and not be misrepresented as something it might be or is not [11].

## 5. Maxwell’s Demon and Second Law Challenges

A demonic being, introduced by Maxwell, to miraculously create thermal non-equilibrium by taking advantage of non-uniform distribution of molecular velocity in equilibrium, and thereby violate the Second law of thermodynamics, has been among the most intriguing and elusive wishful concepts for 150 years now. Maxwell and his followers focused on “effortless gating” a molecule at a time, but overlooked simultaneous interference of other chaotic molecules, while the demon exorcists tried to justify impossible processes with misplaced “compensations” by work of measurements and gate operation, and information storage and memory erasure with entropy generation. It is reasoned phenomenologically and deduced by this author that a Maxwell’s demon operation, against natural forces and without due work effort, is not possible, since it would be against the physics of the chaotic thermal motion, the latter without consistent molecular directional preference for selective timing to be possible [12]. Maxwell’s demon (MD) would have miraculous useful effects, but also some catastrophic consequences.

**Figure 5:**
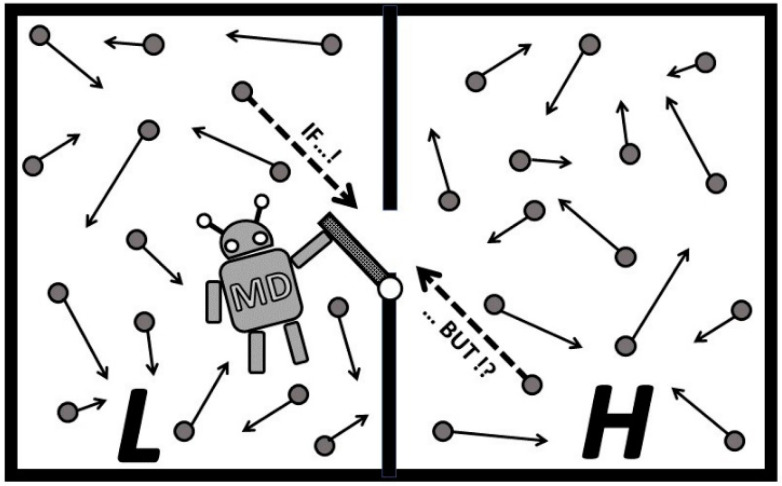
Maxwell’s demon (MD) operates its gate. MD opens the gate to “wishfully pass” a higher speed molecule from *L* to *H* (see dashed arrow line in *L*) and lower speed in reverse. However, considering the chaotic and fast, simultanious molecular thermal motion (most molecules are faster than sound speed), it is probable that the same or even higher speed molecule from *H* will pass back to *L* in that time period (or collide with an oncoming molecule, see dashed arrow line in *H*). Even higher speed molecules may pass back from *H* to *L*, and more probably so if MD was “successful by chance” to separate more high speed molecules into *H*. Therefore, “just opening the gate” would “more equalize than separate” by speed.

The most crucial fact, that the integral, chaotic and simultaneous interactions of all thermal particles on the MD’s operation, has been overlooked, but focus on a single, opportunistic particle motion is emphasized, as if the other thermal particles would not interfere, see Figure 5. Due effort to suppress such forced interference of other thermal particles would amount to required, major “due-work” to establish a macro non-equilibrium, which is independent and in addition to auxiliary “gate-work” of MD to observe molecules and operate a gate. The former, thermodynamic due-work, is unavoidable and substantial, while the latter, MD’s operational work, could be infinitesimally small if the MD’s operation is perfected to near-reversible actions, thus making delusion of the Second law violation.

**Figure 6:**
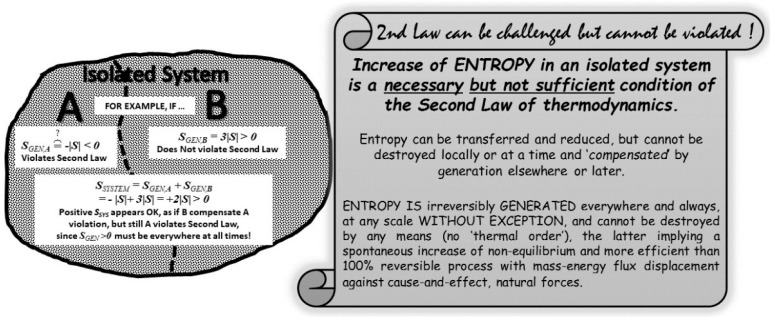
Destruction of entropy is impossible and cannot be “compensated” elsewhere or at later time. “Entropy of an isolated, closed system (or universe) is always increasing”, is a necessary but not sufficient condition of the Second Law of thermodynamics. Entropy cannot be destroyed, locally or at a time, and “compensated” by generation elsewhere or later. It would be equivalent to allow rivers to spontaneously flow uphill and compensate it by more downhill flow elsewhere or later. Entropy is generated everywhere and always, at any scale without exception, and cannot be destroyed by any means at any scale. Impossibility of entropy reduction by destruction should not be confused with local entropy decrease due to entropy outflow with heat [12].

Landauer [13] and his followers, recognizing that the information and storage work suggested by Szilard [14] and his followers is inadequate, introduced additional fallacies to save the Second law. They stated that any MD’s miraculous work gain and related entropy reduction is compensated by follow-up memory information-erasure with entropy generation. However, a fundamental law cannot be selectively violated and then “saved” by compensation elsewhere later, see Figure 6. Entropy cannot be destroyed by MD, locally or at a time, and “compensated” by generation elsewhere or later. It would be equivalent to allow rivers to spontaneously flow uphill and compensate it by more downhill flow later. We cannot pick-and-choose to violate a fundamental law and compensate it later elsewhere. Entropy is generated everywhere and always, at any scale without exception, and cannot be destroyed by any means at any scale [6,11]. Impossibility of entropy reduction by destruction should not be confused with local entropy decrease due to entropy outflow with heat (thermodynamic entropy is associated with thermal motion or heat only).

## 6. Exergy and Entransy, and Beyond

Exergy, as work potential of a system in non-equilibrium with regard to a so-called, reference “dead-state”, is well defined in classical textbooks and references. Here, some selected challenges will be discussed.

**Figure 7:**
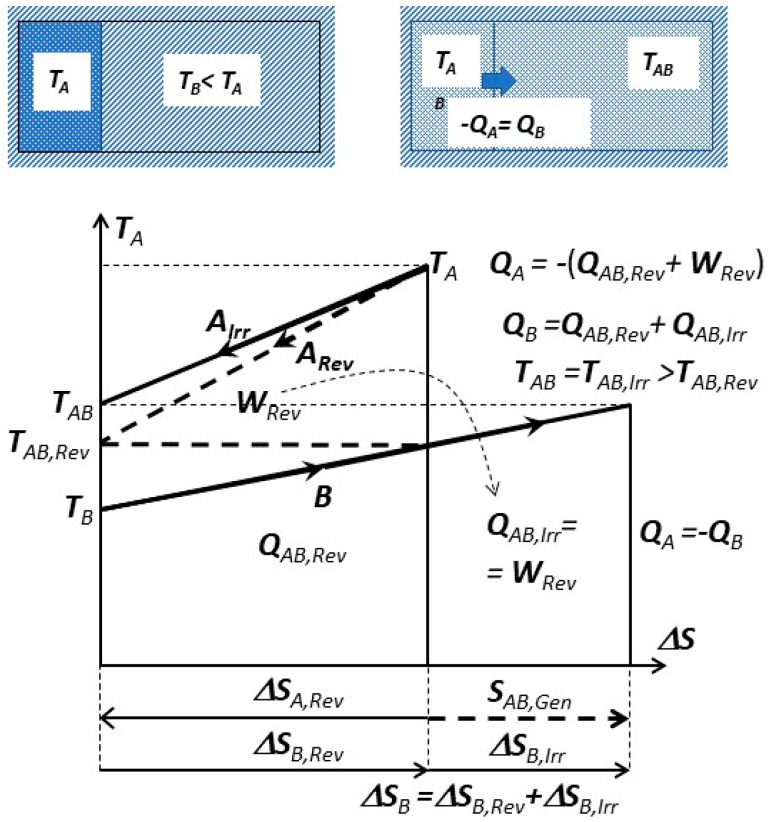
Irreversible (solid lines) versus reversible process (dashed lines) towards mutual equilibrium. System *A* and *B*, each at constant volume, in thermal contact but isolated from the rest of surrounding.

Irreversible versus reversible process towards mutual equilibrium is presented on Figure 7. The isolated, combined system (parts *A* and *B*) is initially at non-equilibrium (*T_B_* < *T_A_*). The mutual work-potential (*W_Rev_*) is fully dissipated with related entropy generation (*S_AB,Gen_*) during the irreversible process leading to mutual equilibrium at *T_AB_* = *T_AB,Irr_*.

(1) $$S_{AB,Gen}=\int_{T_{initial\left(A+B\right)}}^{T_{final\left(AB\right)}}\frac{\delta Q_{Gen}}{T}=m_{A}c_{A}ln\frac{T_{AB}}{T_{A}}+m_{B}c_{B}ln\frac{T_{AB}}{T_{B}}$$

If, during a reversible process, the work-potential is extracted and entropy conserved, it will lead to another mutual equilibrium at *T_AB,Rev_* with entropy changes, but without entropy generation:

(2) $$\Delta S_{A,Rev}=\int_{T_{A}}^{T_{AB,Rev}}\frac{m_{A}c_{A}\cdot dT}{T}=m_{A}c_{A}ln\frac{T_{AB,Rev}}{T_{A}}=-m_{B}c_{B}ln\frac{T_{AB,Rev}}{T_{B}}=-\Delta S_{B,Rev}$$

If the reversible work potential is extracted from the combined system *A + B*, see dashed lines on Figure 7, it will come to a different mutual equilibrium at *T_AB,Rev_* (Equation (2)), smaller than in spontaneous irreversible case at *T_AB_* = *T_AB,Irr_*, since the work potential will dissipate within the combined system instead of being extracted out, i.e.,:

(3) $$T_{AB,Rev}=exp\left[\frac{m_{A}\cdot c_{A}\cdot lnT_{A}+m_{B}\cdot c_{B}\cdot lnT_{B}}{m_{A}\cdot c_{A}+m_{B}\cdot c_{B}}\right]\mathrm{versus}\;T_{AB}=T_{AB,Irr}\;=\frac{m_{A}\cdot c_{A}\cdot T_{A}+m_{B}\cdot c_{B}\cdot T_{B}}{m_{A}\cdot c_{A}+m_{B}\cdot c_{B}}>T_{AB,Rev}$$

Note (on Figure 7) that the mutual work-potential *W_Rev_* and entropy generation *S_AB,Gen_* are not related, and thus not dependent on any reference, surrounding dead-state (*P_o_, T_o_*), since the combined system (*A + B*) is isolated from its surrounding. However, it is capable of producing (extracting) work due to its initial non-equilibrium. That work-potential is completely dissipated (heat generation within) after coming spontaneously (irreversibly) in mutual equilibrium. Actually, the required condition for spontaneous process is the existence of “mutual, non-equilibrium work potential”. The “exergy” is “hypothetical work-potential” if a system reversibly comes to equilibrium by interacting with an arbitrary reference dead-state system (i.e., surrounding). Exergy is useful for comparison, and practical if our systems are coming in equilibrium with such a reference surrounding (i.e., the case with many engineering processes and the Earth’s surroundings, i.e., environment).

Note also that boundary heat transfer (*Q_Bry_*) at finite temperature difference may be considered as reversible at boundary temperature *T_Bry_*, and that the irreversibility takes place within the system when the boundary heat is received at a lower system temperature *T_Sys_*, thus resulting in generated heat and the remaining reversible heat at the system level (heat totality, such as original caloric, conserved). This may be perplexing, since it depends where the irreversibility takes place (whether the temperature gradients are within a layer close to the boundary on the system or surrounding’s sides, or within the system), but if properly accounted for, it will result in the same outcome.

Furthermore, the irreversibility is related to a process, not a system *per se*. For example, if the (sub)system *B* is heated from *T_B_* to *T_AB_*, instead with system *A*, but with another system *B*^+^ with variable temperature, always infinitesimally higher than A’s, then such a process would be reversible (in limit) and without (or infinitesimally small) entropy generation. The *entropy* (a system property) is subtle and so is irreversible *entropy generation* (a process quantity), that it becomes the property after the process is finished.

The irreversible work loss, i.e., work dissipation to generated heat (*W_Loss_* = *W_Diss_* = *Q_Gen_*) and entropy generation (*S_Gen_*) are function of the initial and final process states’ properties only, and not of any other reference dead-state, as it might allude at first, since the combined system (*A* + *B*) is isolated from the surrounding, i.e., it comes to mutual equilibrium, and does not interact with the surrounding.

Similarly, for example, with reference to a surrounding dead-state at *T_o_* & *P_o_*, the exergy of heat *Q*_1_ from a reservoir at temperature *T*_1_ is *E_x_*_1_ *= Q*_1_(1 *− T_o_/T*_1_) and at state 2 would be *E_x_*_2_ *= Q*_2_(1 *− T_o_/T*_2_). It appears that the exergy difference, *E_x_*_1_ *− E_x_*_2_, is a function of *T_o_*. However, for reversible cycle, *Q*_2_*/T*_2_ *= Q*_1_*/T*_1_ (Carnot ratio equality), the relevant quantities are correlated, so the above is reduced to:

*E*_*x*1_ − *E*_*x*2_ = *Q*_1_(1 − *T_o_*/*T*_1_) − *Q*_2_(1 − *T_o_*/*T*_2_) = *Q*_1_(1 − *T_o_*/*T*_1_) − (*Q*_1_·*T*_2_/*T*_1_)(1 − *T_o_*/*T*_2_) = (*Q*_1_/*T*_1_)(*T*_1_ − *T*_2_) (4)

This is an interesting and deceptive outcome: the change of exergy of heat is not the function of dead-state temperature *T_o_*. Therefore, it is unnecessary to use exergy (which is based on a hypothetical reference, surrounding dead-state, *T_o_*, *P_o_*). Furthermore, for isolated processes without interaction with the surrounding, it may be inappropriate, to use exergy difference, since actual work-loss is relative to mutual equilibrium state reached between the two isolated sub/systems, as demonstrated above (there is no *T_o_* and *P_o_* in the above expressions). It may be the case for all thermal processes with no net-entropic, nor net-volumetric interactions with the surrounding. It requires further discussions and clarifications.

Entropy is generated when work potential is lost (i.e., dissipated) into “generated heat transfer” (randomly equi-partitioned) into the thermal energy at given absolute temperature within the space occupied by the system, or when expansion (elastic) work potential is lost (i.e., energy randomly redistributed within enlarged volume, as in the free expansion, instead of being extracted as work (volume displacement against the surrounding or load pressure). Therefore, heat is thermal energy transfer due to temperature gradient, while work is other than thermal energy transfer due to other energy-potential gradients, such as pressure, elevation, voltage, gravity, electro-magnetic, electro-chemical, etc.

The entropy unit is not “exactly the same” as for the specific heat, since entropy increase at constant volume is equal to the thermal energy increase per absolute temperature level (important), as opposed to per temperature difference for specific heat at constant volume. Entropy also increases with volume increase at constant temperature and during adiabatic expansion unless the latter is reversible (i.e., isentropic; entropy increase due to volume expansion is balanced with equal decrease due to work extraction and the corresponding internal energy reduction). Therefore, during reversible change of volume (mechanical, not thermal process) there is no change of entropy due to change of volume, but only due to boundary heat transfer if any.

A new property, based on physical analogy between electrical conduction, represented by the Ohm’s law (the electrical charge *Q_v**e**_ = I·t = E_e_*/*V*), and heat conduction, is introduced from *Q_v**h**_ = Z_tr_*/*T = E_vh_*/*T = G*/*T* (as concept-in-general, if electrical charge and heat are transferred at constant voltage and temperature, respectively), thus, in principle, defining a new physical quantity, “entransy” [15,16,17,18], i.e.,:

(5) $$G\equiv E_{vh}\equiv Z_{tr}=Q_{vh}T$$

Note that designation of “stored heat” (*Q_v**h**_*), has been utilized for stored heat as thermal energy within the material system, For the new quantity, entransy different symbols have been used in subsequent publications.

The entransy has been defined as a state property, as a function of ”stored heat”. Although the real system specific heat is function of temperature, the entransy has been regrettably and unnecessarily restricted for constant specific heat systems. It is suggested that entransy be defined instead, as integral quantity for variable specific heat for incompressible systems and/or constant volume processes *C_v_* = *f*(*P*, *T*) ≈ *f*(*T*), i.e.,

*G* = ∫*MC_v_TdT* (6)

**Figure 8:**
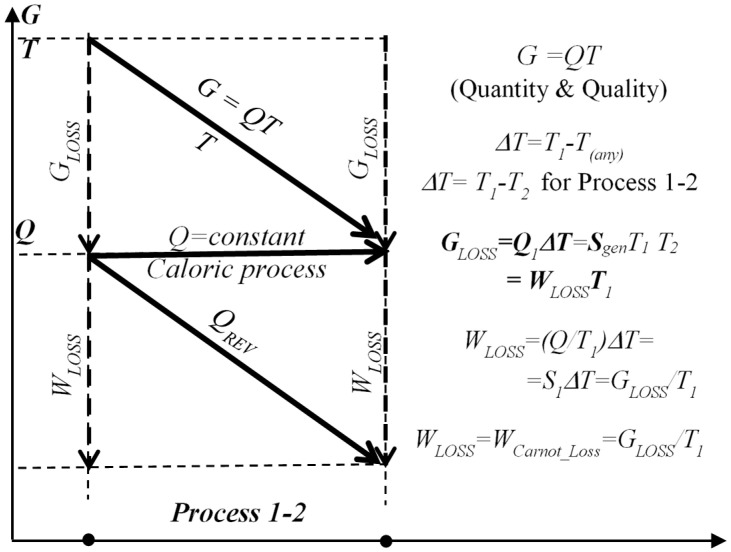
Correlation between entransy *G*, reversible heat *Q_REV_*, entransy dissipation or loss *G_LOSS_*, and Carnot work-potential loss *W_LOSS_*, during 1-D steady-state heat conduction caloric process 1–2 with conserved heat transfer *Q*.

Correlations between entransy *G*, reversible heat *Q_REV_*, entransy dissipation or loss *G_LOSS_*, and Carnot work-potential loss *W_LOSS_* is presented on Figure 8.

Furthermore, the “entransy of work”, *G_W_*, is also essential to be defined for processes when thermal heat is converted to work, such as in heat engines. The work entransy could be defined using the entransy balance for reversible, Carnot cycle relationship, and considering that there is no entransy loss in an ideal reversible process, i.e., *G_IN_ = G_OUT_* or *G*_1_ *= G_W_ + G*_2_ (notation in [18]):

*G_W_* = *G*_1_ − *G*_2_ = *G*_1_(1 − (*T*_2_^2^/*T*_1_^2^)) or *dG_W_* = (1 − (*T*_2_^2^/*T*_1_^2^))·*dG*_1_ (7)

In another publication [19], the work entransy, *G_W_*, was derived by algebraic manipulation as *G_W_ = WT*_1_ *=* (*Q*_1_ *− Q*_2_)*T*_1_ *= Q*_1_(*T*_1_ *− T*_2_). However, this definition is not appropriate since it does not satisfy condition that entransy loss is zero for reversible processes. There is a need for further interpretations of the entransy concept and possible refinements [18].

## 7. Challenges and Concluding Remarks

Starting from Clausius till nowadays, the obvious but in general not quantified thermal energy, is “lumped” into well-quantified internal energy, provided in Thermodynamic data tables. Some (or many) argue that subtle “thermal energy” is not definable, but the “internal thermal energy” is manifested as heat transfer due to temperature difference. It is argued here and elsewhere (related manuscripts being finalized by this author) that heat (and anything else for that matter) could be transferred only if it exists as stored quantity in kind, in the first place. It is self-evident in caloric processes and quantified by the caloric quantities that the “thermal energy” is stored heat (directly related to the system heat capacity), *U_Th_* ≡ *Q_Stored_*, and heat is the thermal energy transfer, *Q* ≡ *U_Th,transfer_*, [10]. 

The Second law is not about disorder and probability *per se* (or any other math or physics “tools” *per se* used to describe it), but about spontaneous, forced-tendency (natural process-forcing displacement) of mass-energy redistribution in certain, irreversible direction (process driving force), from higher to lower energy-potential (mass-energy density in space). Spontaneity implies forced-directionality and in turn irreversibility. No spontaneous, irreversible process could ever be completely reversed or undone. For example, the driving force for the life and processes on Earth is the irreversible dissipation of energy from the Sun.

It is hard to believe that a serious scientist, who truly comprehends the Second law and its essence, would challenge the concept based on incomplete and elusive facts. Sometimes, highly respected scientists in their fields, do not fully comprehend the essence of the Second law of thermodynamics. The Second law “challengers” need to demonstrate and quantify destruction of entropy to challenge the universal validity of the Second law. It has been reasoned and thus proven that destruction of entropy, i.e., violation of the Second law, is against the forced tendency of natural processes and thus impossible, leaving “*No Hope*” for the challengers [11]. After all, the “Wishful Maxwell’s demon” could not be realized since its introduction in 1867. Therefore, before “the Second law violation” claims are stated, the reliable criteria for the Second law violation, including proper definition and evaluation of entropy, should be established based on full comprehension of the fundamental natural Laws.

A critical treatise of “Entransy concept and controversies” within elusive thermal landscape, has been given recently by this author [18]. Regardless of entransy redundancy, being derived from other physical quantities, it does not diminish its usefulness and uniqueness in thermal analysis and optimization. Despite the need for further development and clarifications of the new concept, it is argued in [18] that the entransy, due to its unique nature, may contribute to better comprehension of often obscured thermal phenomena. It would be unjust and premature, based on limited and subjective claims, to discredit entransy as if the “already established” concepts and methodologies are perfect, and do not need alternatives and innovations, as if further progress is not needed.

As the fundamental laws of nature and thermodynamics are expended from simple systems in physics and chemistry, to different space and time scales and to much more complex systems in biology, life and intelligent processes, there are more challenges to be comprehended and understood.
